# *N*-(2-Chloro­phen­yl)succinimide

**DOI:** 10.1107/S1600536810010457

**Published:** 2010-03-27

**Authors:** B. S. Saraswathi, B. Thimme Gowda, Sabine Foro, Hartmut Fuess

**Affiliations:** a Department of Chemistry, Mangalore University, Mangalagangotri 574 199, Mangalore, India; b Institute of Materials Science, Darmstadt University of Technology, Petersenstrasse 23, D-64287 Darmstadt, Germany

## Abstract

In the title compound, C_10_H_8_ClNO_2_, the dihedral angle between the aromatic benzene ring and the imide segment is 69.5 (1)°. In the crystal structure, mol­ecules are linked by very weak C—H⋯π inter­actions along the [001] direction.

## Related literature

For our study of the effect of ring and side-chain substitutions on the structures of this class of compounds, see: Gowda *et al.* (2007 ▶); Saraswathi *et al.* (2010**a* ▶,b* ▶).
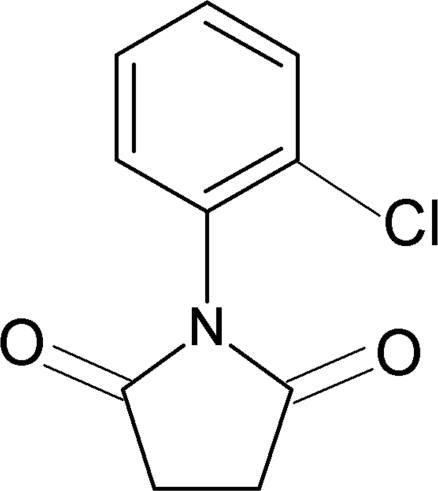


## Experimental

### 

#### Crystal data

C_10_H_8_ClNO_2_
*M* *_r_* = 209.62
Orthorhombic, 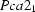

*a* = 10.616 (1) Å
*b* = 11.191 (2) Å
*c* = 8.220 (1) Å
*V* = 976.6 (2) Å^3^
*Z* = 4
Mo *K*α radiation
μ = 0.36 mm^−1^
*T* = 299 K
0.50 × 0.48 × 0.40 mm

#### Data collection

Oxford Diffraction Xcalibur diffractometer with a Sapphire CCD detector
Absorption correction: multi-scan (*CrysAlis RED*; Oxford Diffraction, 2009 ▶) *T*_min_ = 0.840, *T*_max_ = 0.869
2417 measured reflections
1600 independent reflections
1500 reflections with *I* > 2σ(*I*)
*R*_int_ = 0.008

#### Refinement

*R*[*F* ^2^ > 2σ(*F* ^2^)] = 0.025
*wR*(*F* ^2^) = 0.072
*S* = 1.04
1600 reflections
128 parameters
1 restraint
H-atom parameters constrained
Δρ_max_ = 0.19 e Å^−3^
Δρ_min_ = −0.23 e Å^−3^
Absolute structure: Flack (1983 ▶), 525 Friedel pairs
Flack parameter: 0.01 (7)

### 

Data collection: *CrysAlis CCD* (Oxford Diffraction, 2009 ▶); cell refinement: *CrysAlis RED* (Oxford Diffraction, 2009 ▶); data reduction: *CrysAlis RED*; program(s) used to solve structure: *SHELXS97* (Sheldrick, 2008 ▶); program(s) used to refine structure: *SHELXL97* (Sheldrick, 2008 ▶); molecular graphics: *PLATON* (Spek, 2009 ▶); software used to prepare material for publication: *SHELXL97*.

## Supplementary Material

Crystal structure: contains datablocks I, global. DOI: 10.1107/S1600536810010457/bx2270sup1.cif

Structure factors: contains datablocks I. DOI: 10.1107/S1600536810010457/bx2270Isup2.hkl

Additional supplementary materials:  crystallographic information; 3D view; checkCIF report

## Figures and Tables

**Table 1:** C—H⋯π inter­action geometry (Å, °) *Cg*1 is the centroid of the C1–C6 ring.

D— H⋯ A	D— H	H⋯ A	D⋯ A	D— H⋯ A
C3—H3⋯*Cg*1^i^	0.93	2.93	3.76 (2)	149

Symmetry code: (i) −*x* + 1, −*y*, *z* + 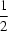.

**Fig. 1.**
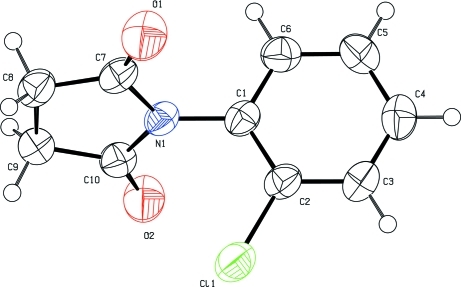
Molecular structure of the title compound, showing the atom labelling scheme. The displacement ellipsoids are drawn at the 50% probability level. The H atoms are represented as small spheres of arbitrary radii.

**Fig. 2.**
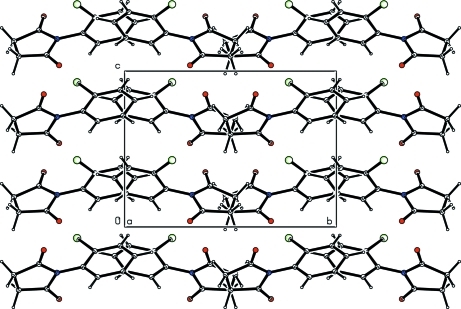
Molecular packing of the title compound.

## supplementary crystallographic information

### Comment

The amide moiety is an important constituent of many biologically significant compounds. As a part of studying the effect of ring and side chain substitutions on the structures of this class of compounds (Gowda *et al.*, 2007; Saraswathi *et al.*, 2010*a,b*), the crystal structure of *N*,*N*-(2-chlorophenyl)succinimide has been determined (Fig. 1). In the structure of the title compound, C_10_H_8_ClNO_2 _, the molecule is non-planar with the benzene and pyrrolidine rings tilted by 69.5 (1)° with respect to one another. In the crystal structure, the molecules are linked by weak C—H···π interactions.

### Experimental

The solution of succinic anhydride (2.5 g) in toluene (25 ml) was treated dropwise with the solution of 2-chloroaniline (2.5 g) also in toluene(20 ml) with constant stirring. The resulting mixture was stirred for about one hour and set aside for an additional hour at room temperature for completion of the reaction. The mixture was then treated with dilute hydrochloric acid to remove the unreacted 2-chloroaniline. The resultant solid *N*-(2-chlorophenyl)succinamic acid was filtered under suction and washed thoroughly with water to remove the unreacted succinic anhydride and succinic acid. It was recrystallized to constant melting point from ethanol. *N*-(2-chlorophenyl)succinamic acid was then heated for 2 hours and then allowed to cool slowly to room temperature to get crystals of *N*-(2-chlorophenyl)succinimide. The purity of the compound was checked and characterized by its infrared spectra.

The prism like colourless single crystals of the compound used in X-ray diffraction studies were grown in ethanolic solution by a slow evaporation at room temperature.

### Refinement

The H atoms were positioned with idealized geometry using a riding model with C—H in the range 0.93–0.97 Å. U_iso_(H) values were set equal to 1.2U_eq_(parent atom).

### Crystal data

**Table:**

C_10_H_8_ClNO_2_	*F*(000) = 432
*M**_r_* = 209.62	*D*_x_ = 1.426 Mg m^−^^3^
Orthorhombic, *P**c**a*2_1_	Mo *K*α radiation, λ = 0.71073 Å
Hall symbol: P 2c -2ac	Cell parameters from 1700 reflections
*a* = 10.616 (1) Å	θ = 2.6–27.7°
*b* = 11.191 (2) Å	µ = 0.36 mm^−^^1^
*c* = 8.220 (1) Å	*T* = 299 K
*V* = 976.6 (2) Å^3^	Prism, colourless
*Z* = 4	0.50 × 0.48 × 0.40 mm

### Data collection

**Table:**

Oxford Diffraction Xcalibur diffractometer with a Sapphire CCD detector	1600 independent reflections
Radiation source: fine-focus sealed tube	1500 reflections with *I* > 2σ(*I*)
graphite	*R*_int_ = 0.008
Rotation method data acquisition using ω and φ scans	θ_max_ = 26.4°, θ_min_ = 2.6°
Absorption correction: multi-scan (*CrysAlis RED*; Oxford Diffraction, 2009)	*h* = −6→13
*T*_min_ = 0.840, *T*_max_ = 0.869	*k* = −13→8
2417 measured reflections	*l* = −7→10

### Refinement

**Table:**

Refinement on *F*^2^	Hydrogen site location: inferred from neighbouring sites
Least-squares matrix: full	H-atom parameters constrained
*R*[*F*^2^ > 2σ(*F*^2^)] = 0.025	*w* = 1/[σ^2^(*F*_o_^2^) + (0.0446*P*)^2^ + 0.1451*P*] where *P* = (*F*_o_^2^ + 2*F*_c_^2^)/3
*wR*(*F*^2^) = 0.072	(Δ/σ)_max_ < 0.001
*S* = 1.04	Δρ_max_ = 0.19 e Å^−^^3^
1600 reflections	Δρ_min_ = −0.22 e Å^−^^3^
128 parameters	Extinction correction: *SHELXL97* (Sheldrick, 2008), Fc^*^=kFc[1+0.001xFc^2^λ^3^/sin(2θ)]^-1/4^
1 restraint	Extinction coefficient: 0.036 (3)
Primary atom site location: structure-invariant direct methods	Absolute structure: Flack (1983), 525 Friedel pairs
Secondary atom site location: difference Fourier map	Flack parameter: 0.01 (7)

### Special details

**Table:**

Experimental. (CrysAlis RED; Oxford Diffraction, 2009) Empirical absorption correction using spherical harmonics, implemented in SCALE3 ABSPACK scaling algorithm.
Geometry. All esds (except the esd in the dihedral angle between two l.s. planes) are estimated using the full covariance matrix. The cell esds are taken into account individually in the estimation of esds in distances, angles and torsion angles; correlations between esds in cell parameters are only used when they are defined by crystal symmetry. An approximate (isotropic) treatment of cell esds is used for estimating esds involving l.s. planes.
Refinement. Refinement of *F*^2^ against ALL reflections. The weighted *R*-factor wR and goodness of fit *S* are based on *F*^2^, conventional *R*-factors *R* are based on *F*, with *F* set to zero for negative *F*^2^. The threshold expression of *F*^2^ > σ(*F*^2^) is used only for calculating *R*-factors(gt) etc. and is not relevant to the choice of reflections for refinement. *R*-factors based on *F*^2^ are statistically about twice as large as those based on *F*, and *R*- factors based on ALL data will be even larger.

### Fractional atomic coordinates and isotropic or equivalent isotropic displacement parameters (Å^2^)

**Table:**

	*x*	*y*	*z*	*U*_iso_*/*U*_eq_	
Cl1	0.40098 (5)	0.22415 (4)	0.92831 (9)	0.0664 (2)	
O1	0.77333 (15)	0.38146 (14)	0.8513 (2)	0.0691 (5)	
O2	0.42907 (15)	0.30911 (14)	0.5374 (2)	0.0625 (4)	
N1	0.60155 (12)	0.31896 (14)	0.70608 (18)	0.0386 (4)	
C1	0.61026 (16)	0.19473 (17)	0.7438 (2)	0.0388 (4)	
C2	0.52257 (16)	0.14047 (17)	0.8445 (3)	0.0438 (4)	
C3	0.5306 (2)	0.02002 (18)	0.8789 (3)	0.0507 (5)	
H3	0.4708	−0.0160	0.9455	0.061*	
C4	0.6275 (2)	−0.04682 (19)	0.8141 (3)	0.0529 (5)	
H4	0.6333	−0.1279	0.8375	0.063*	
C5	0.71622 (19)	0.00642 (19)	0.7144 (3)	0.0540 (5)	
H5	0.7817	−0.0388	0.6712	0.065*	
C6	0.70742 (17)	0.12731 (18)	0.6789 (3)	0.0477 (5)	
H6	0.7668	0.1631	0.6116	0.057*	
C7	0.68670 (17)	0.40365 (17)	0.7621 (2)	0.0437 (4)	
C8	0.65005 (18)	0.52249 (17)	0.6916 (3)	0.0481 (5)	
H8A	0.7161	0.5527	0.6211	0.058*	
H8B	0.6348	0.5802	0.7773	0.058*	
C9	0.53041 (17)	0.49950 (18)	0.5953 (3)	0.0495 (5)	
H9A	0.4599	0.5421	0.6428	0.059*	
H9B	0.5405	0.5251	0.4833	0.059*	
C10	0.50941 (17)	0.36713 (18)	0.6038 (3)	0.0429 (4)	

### Atomic displacement parameters (Å^2^)

**Table:**

	*U*^11^	*U*^22^	*U*^33^	*U*^12^	*U*^13^	*U*^23^
Cl1	0.0671 (4)	0.0458 (3)	0.0864 (4)	−0.0093 (2)	0.0334 (3)	−0.0081 (3)
O1	0.0631 (9)	0.0671 (10)	0.0771 (11)	−0.0155 (8)	−0.0345 (9)	0.0026 (8)
O2	0.0574 (8)	0.0553 (9)	0.0749 (11)	−0.0085 (7)	−0.0267 (8)	0.0040 (8)
N1	0.0364 (7)	0.0401 (8)	0.0394 (9)	−0.0075 (6)	−0.0032 (7)	0.0028 (7)
C1	0.0378 (8)	0.0416 (9)	0.0370 (10)	−0.0080 (7)	−0.0070 (7)	0.0018 (8)
C2	0.0422 (9)	0.0431 (10)	0.0461 (11)	−0.0087 (8)	0.0032 (8)	−0.0038 (8)
C3	0.0550 (11)	0.0439 (10)	0.0531 (13)	−0.0117 (9)	0.0033 (10)	0.0039 (9)
C4	0.0633 (12)	0.0419 (10)	0.0535 (12)	−0.0019 (9)	−0.0108 (10)	0.0058 (10)
C5	0.0504 (11)	0.0549 (11)	0.0565 (13)	0.0114 (9)	−0.0025 (10)	0.0015 (10)
C6	0.0408 (9)	0.0567 (11)	0.0457 (11)	−0.0003 (8)	−0.0001 (9)	0.0052 (10)
C7	0.0433 (9)	0.0465 (10)	0.0414 (10)	−0.0120 (8)	0.0020 (8)	−0.0039 (8)
C8	0.0518 (10)	0.0430 (10)	0.0495 (11)	−0.0090 (9)	0.0068 (9)	−0.0039 (9)
C9	0.0443 (11)	0.0441 (10)	0.0601 (12)	0.0007 (8)	0.0014 (9)	0.0021 (10)
C10	0.0401 (9)	0.0447 (10)	0.0439 (10)	−0.0024 (8)	0.0001 (9)	0.0005 (8)

### Geometric parameters (Å, °)

**Table:**

Cl1—C2	1.737 (2)	C4—H4	0.9300
O1—C7	1.202 (2)	C5—C6	1.387 (3)
O2—C10	1.203 (2)	C5—H5	0.9300
N1—C7	1.388 (2)	C6—H6	0.9300
N1—C10	1.398 (2)	C7—C8	1.502 (3)
N1—C1	1.427 (2)	C8—C9	1.519 (3)
C1—C6	1.385 (3)	C8—H8A	0.9700
C1—C2	1.386 (3)	C8—H8B	0.9700
C2—C3	1.380 (3)	C9—C10	1.500 (3)
C3—C4	1.379 (3)	C9—H9A	0.9700
C3—H3	0.9300	C9—H9B	0.9700
C4—C5	1.383 (3)		
			
C7—N1—C10	113.08 (16)	C5—C6—H6	120.0
C7—N1—C1	123.41 (15)	O1—C7—N1	124.02 (19)
C10—N1—C1	123.47 (14)	O1—C7—C8	128.07 (18)
C6—C1—C2	119.44 (18)	N1—C7—C8	107.91 (16)
C6—C1—N1	119.70 (16)	C7—C8—C9	105.54 (15)
C2—C1—N1	120.86 (17)	C7—C8—H8A	110.6
C3—C2—C1	120.59 (18)	C9—C8—H8A	110.6
C3—C2—Cl1	119.42 (15)	C7—C8—H8B	110.6
C1—C2—Cl1	119.99 (15)	C9—C8—H8B	110.6
C4—C3—C2	119.79 (19)	H8A—C8—H8B	108.8
C4—C3—H3	120.1	C10—C9—C8	105.50 (16)
C2—C3—H3	120.1	C10—C9—H9A	110.6
C3—C4—C5	120.20 (19)	C8—C9—H9A	110.6
C3—C4—H4	119.9	C10—C9—H9B	110.6
C5—C4—H4	119.9	C8—C9—H9B	110.6
C4—C5—C6	119.92 (19)	H9A—C9—H9B	108.8
C4—C5—H5	120.0	O2—C10—N1	124.13 (18)
C6—C5—H5	120.0	O2—C10—C9	128.10 (19)
C1—C6—C5	120.06 (18)	N1—C10—C9	107.77 (16)
C1—C6—H6	120.0		
			
C7—N1—C1—C6	−69.2 (2)	C4—C5—C6—C1	0.2 (3)
C10—N1—C1—C6	108.2 (2)	C10—N1—C7—O1	−179.95 (19)
C7—N1—C1—C2	110.9 (2)	C1—N1—C7—O1	−2.3 (3)
C10—N1—C1—C2	−71.7 (2)	C10—N1—C7—C8	−0.1 (2)
C6—C1—C2—C3	−0.8 (3)	C1—N1—C7—C8	177.56 (16)
N1—C1—C2—C3	179.19 (17)	O1—C7—C8—C9	−177.3 (2)
C6—C1—C2—Cl1	179.40 (15)	N1—C7—C8—C9	2.9 (2)
N1—C1—C2—Cl1	−0.7 (2)	C7—C8—C9—C10	−4.4 (2)
C1—C2—C3—C4	0.8 (3)	C7—N1—C10—O2	177.3 (2)
Cl1—C2—C3—C4	−179.36 (17)	C1—N1—C10—O2	−0.3 (3)
C2—C3—C4—C5	−0.3 (3)	C7—N1—C10—C9	−2.8 (2)
C3—C4—C5—C6	−0.2 (3)	C1—N1—C10—C9	179.55 (16)
C2—C1—C6—C5	0.2 (3)	C8—C9—C10—O2	−175.7 (2)
N1—C1—C6—C5	−179.71 (18)	C8—C9—C10—N1	4.4 (2)

### Table 1 C—H···π interaction geometry ( Å, ° )

Cg1 is the centroid of the C1–C6 ring.

**Table:**

D— H··· A	D— H	H··· A	D··· A	D— H··· A
C3—H3···Cg1^i^	0.93	2.93	3.76 (2)	149

Symmetry code: (i) -x+1, -y, z+1/2.
